# Effect of CPAP Therapy on Serum Lipids and Blood Pressure in Patients with Obstructive Sleep Apnea Syndrome

**Published:** 2019-02

**Authors:** Abdolah Asgari, Forogh Soltaninejad, Ziba Farajzadegan, Babak Amra

**Affiliations:** 1 Department of Internal Medicine, Isfahan University of Medical Sciences, Isfahan, Iran,; 2 Bamdad Respiratory and Sleep Research Center, Pulmonary Unit, Department of Internal Medicine, Isfahan University of Medical Sciences, Isfahan, Iran,; 3 Department of Community and Preventive Medicine, Isfahan University of Medical Sciences, Isfahan, Iran.

**Keywords:** Continuous positive airway pressure, Blood Pressure, Serum Lipids, Obstructive Sleep Apnea Syndrome

## Abstract

**Background:**

Most of patients with Obstructive Sleep Apnea (OSA) are at risk of metabolic syndrome. The treatment of choice for OSA patients is the Continuous Positive Airway Pressure (CPAP). Reports about the effect of CPAP on metabolic parameters are controversial. So, we aimed to evaluate the effect of CPAP therapy on blood pressure, Fasting Blood Sugar (FBS), and serum lipids including: Triglycerides (TG), total cholesterol (Chol), High Density Lipoproteins (HDL), and Low Density Lipoproteins (LDL).

**Materials and Methods:**

This study included 35 OSA patients. Initially, their baseline demographic and clinical characteristics were recorded. Then, the patients underwent CPAP therapy on average 4–7 hours for 8 weeks. The level of FBS, TG, Cholesterol, HDL, LDL, and blood pressure were measured and recorded before and after treatment. Finally, the collected data were analyzed with SPSS version 22.

**Results:**

This study included 35 OSA patients [24 men (68.6%)], with mean age of 45.58±8.02 years. Results indicated that CPAP therapy led to decrease of 9.76 and 3.49 mmHg in systolic and diastolic blood pressures, respectively. Also, LDL decreased to 6.27mg/dl and HDL increased to 0.75 mg/dl (P<0.001) with treatment. The changes of other variables were not significant (P>0.05).

**Conclusion:**

Treatment of OSA with CPAP has beneficial effects on blood pressure and some items of lipid profile. Regarding the importance of metabolic disturbances in OSA complications, choosing an appropriate treatment for OSA patients can play an important role in improvement of patients status as well as prevention of these complications.

## INTRODUCTION

In recent decades sleep disordered breathing, its clinical outcomes and high outbreak have been increasingly considered as a major health problem (1). Obstructive Sleep Apnea (OSA) syndrome can be recognized through frequently recurrence of upper airway obstruction during sleep. It can affect more than 5% of adults (2). About 13% of men and 6% of women, aged 30–70 years have been reported with the mild-to-moderate OSA (3). The syndrome can be remained unknown for long period because these breathing disorders occur over night, while their consequences reflect in individual performance and lead to reduced quality of life, increased sleep arousals, oxygen desaturation and daily sleepiness as well as cognitive disorders (4–6).

According to literature, impaired High Density Lipoproteins (HDL) function and oxidized Low Density Lipoproteins (LDL) levels are more common in OSA patients compared to controls (7). Also, OSA patients are often suffering from metabolic disorders including obesity, insulin resistance, hypertension, dyslipidemia, hyperglycemia and increased Fasting Blood Sugar (FBS) (8). Also, these are generally identified as metabolic syndrome and OSA may be considered as a manifestation of metabolic syndrome (9).

The night Polysomnography (PSG) is the gold standard test to diagnose OSA. In order to evaluate OSA, recording of parameters through PSG including Electroencephalography (EEG), Electrooculography (EOG), Electrocardiography (ECG), oximetry and the measurements of airflow as well as respiratory activities, is done (10).

Continuous Positive Airway Pressure (CPAP) therapy is the main and selective therapy for OSA and known as the first-line treatment (11). The CPAP therapy can help resolve respiratory airway obstructions, daily sleepiness as well as improvement in quality of life (12, 13). The CPAP therapy may affect serum lipids positively (14–16). Recently, it has been considered as a hypothesis that resolving recurrent hypoxia and sympathetic stimulation can result in improved lipid profile (17).

Numerous studies have reported decreased systolic and diastolic blood pressure during CPAP (18, 19), although there are conflict results suggesting the effect of CPAP therapy on other metabolic syndrome components such as insulin resistance (20) and lipid profile (21).

Hence, the effect of CPAP therapy on metabolic syndrome components in OSA patients has remained unclear. Taking into account high prevalence of OSA in adults, recognized association between metabolic syndrome components and sleep disorders of breathing as well as various results of CPAP studies; the current study aimed to evaluate the effect of CPAP therapy on blood glucose and serum lipids in OSA patients.

## MATERIALS AND METHODS

In this cross sectional study, we initially included all patients referred to Bamdad Respiratory and Sleep Research Center in Isfahan during March 2017 to September 2017 for evaluation of OSA. Using Cochran's sample size formula to calculate the optimal number of sampling with 95% confidence interval, a power of the test of 80%, OSA prevalence of 0.35 and the type I error rate of 0.05, we estimated a sample size of 35.

Inclusion criteria included: age ≥18 years, Apnea–Hypopnea Index (AHI) >5, having consent to participate in the study, and agree with using CPAP therapy as the main treatment (if approved by physician). The patients with inherited hypercholesterolemia or hyperglycemia, or those who had taken antihypertensive or lipid-lowering drugs within the recent month were excluded. All patients were highly suspicious of OSA based on STOP-Bang and Epworth Sleepiness Scale (ESS) questionnaires. After screening, standard overnight-attended PSG was done using polysomnography device (SOMNOmedics GmbH, Randersacker, Germany). In standard polysomnography EEG, ECG, EOG, chin electromyogram, oronasal airflow, oxygen saturation by pulse oximetry, thoracic, abdominal and leg movements were recorded. Scoring was done according to American Academy of Sleep Medicine (AASM) 2015 guideline.

Based on AHI in PSG, patients with OSA are categorized to mild (5<AHI≤15), moderate (15<AHI≤30), and severe (AHI>30).

Patients with OSA (AHI>5) were assigned to the study using simple randomization. We recorded their demographic characteristics. Clinical parameters including blood pressure, FBS, Triglycerides (TG), Cholesterol (Chol), HDL, and LDL were measured and recorded.

After titration for at least 4 hours including supine position and Rapid Eye Movement (REM) stage, all OSA patients underwent CPAP therapy and trained on how to use the device. They were asked to use CPAP therapy, on average 4–7 hours per day for 8 weeks and afterwards they should present again at the center to evaluate their lipid profile, FBS, and blood pressure.

### Statistical analysis

Finally, the collected data were analyzed with SPSS (ver. 22). Qualitative and quantitative data were expressed as frequencies (percentages) and Mean ± SD (standard deviation). Given the findings of Kolmogorov–Smirnov test rejected normality of the distribution, we applied Wilcoxon test to compare the effect of CPAP on study parameters. In all analyses, we used a significant level of <0.05.

### Ethical consideration

This research project was approved scientifically by the Research Committee of Isfahan University of Medical Sciences and ethically by the Ethic Committee of the University (Ethic code 395718). Also, it was registered in the Iranian Registration System with IRCT20170826038N16 before starting participant recruitment.

## RESULTS

The current study included 35 OSA patients [24 men (68.6%)], with mean age 45.58±8.02 years, and mean AHI 45.57±17.51 events/h. The frequencies of patients with moderate and severe OSA were 20 and 80%, respectively. Also the mean duration of CPAP use was 4.70±0.96 hours per day (Table 1).

**Table 1.** The basic characteristics of OSA patients

**Characteristics**		N(%)^†^ or Mean(SD) ^*^
**Age; year**		45.58(8.02)^*^
**Sex**	**Male**	24(68.6)^†^
	**Female**	11(31.4)^†^
**BMI; kg/m^2^**		30.17(6.79)^*^
	**<30 kg/m^2^**	18(51.4)^†^
	**≥30 kg/m^2^**	17(48.6)^†^
**AHI; events/h**		45.57(17.51)^*^
	**Mild OSA**	0(0)^†^
	**Moderate OSA**	7(20)^†^
	**Severe OSA**	28(80)^†^
**Duration of CPAP use; hours**		4.70±0.96

AHI group: Mild OSA (5≤AHI<15 events/h); moderate OSA (15<AHI≤30 events/h); and severe OSA (AHI > 30 events/h).

The weight of the patients before and after the treatment were 80.66±15.41 and 80.01±18.68 kg, respectively (P=0.784). Although mean systolic and diastolic blood pressures and mean LDL showed a significant decrease and mean HDL showed a significant increase post-CPAP compared to pre-CPAP (P<0.001), no significant change was observed in other study parameters including FBG, TG, and Chol (P>0.05). Comparison of OSA patients with different severity showed that overall changes in blood pressure and lipid parameters were greater in severe OSA patients than moderate OSA patients, except for total cholesterol (Table 2).

**Table 2.** Comparison of blood and lipid parameters in OSA patients, pre and post CPAP

**Variables**		**Before CPAP**	**After CPAP**	**P value**
**Systolic Blood Pressure (mmHg)**	**Total**	124.18±14.87	114.42±12.21	<0.001
	**Moderate OSA**	104.58±6.69	98.44±8.58	0.006
	**Severe OSA**	129.08±12.01	118.42±9.41	<0.001
**Diastolic Blood Pressure (mmHg)**	**Total**	82.46±10.25	78.97±9.50	<0.001
	**Moderate OSA**	69.72±5.22	67.19±5.22	0.014
	**Severe OSA**	85.64±8.58	81.92±7.97	<0.001
**Fasting blood glucose (mg/dl)**	**Total**	120.32±16.57	118.79±15.89	0.061
	**Moderate OSA**	99.07±4.28	97.51±5.63	0.183
	**Severe OSA**	125.64±13.98	119.86±13.33	0.081
**Triglycerides (mg/dl)**	**Total**	162.18±21.14	158.85±19.93	0.078
	**Moderate OSA**	136.02±10.45	1426.44±19.18	0.086
	**Severe OSA**	168.72±17.80	160.95±15.15	0.053
**Total cholesterol (mg/dl)**	**Total**	326.65±17.13	335.94±29.85	0.105
	**Moderate OSA**	301.71±14.20	337.31±28.54	0.013
	**Severe OSA**	332.88±11.11	335.60±30.67	0.650
**HDL (mg/dl)**	**Total**	34.02±2.18	34.77±2.34	<0.001
	**Moderate OSA**	32.96±1.96	33.68±2.41	0.030
	**Severe OSA**	34.28±2.19	35.04±2.28	<0.001
**LDL (mg/dl)**	**Total**	119.52±15.48	113.25±17.56	<0.001
	**Moderate OSA**	100.81±7.70	92.95±3.48	0.011
	**Severe OSA**	124.19±13.24	118.32±15.89	<0.001

**Figure 1.**
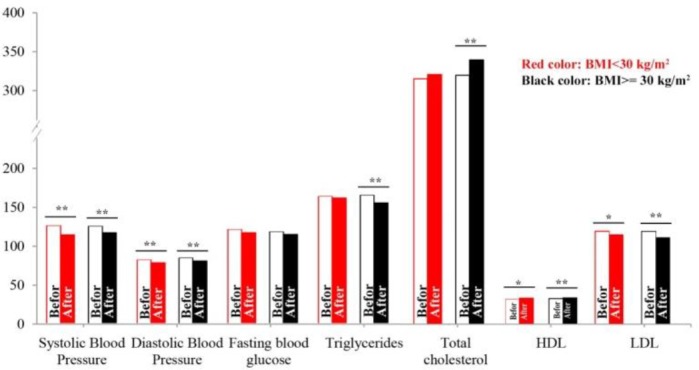
Comparison of blood pressure, FBS and lipid parameters in OSA patients in terms of BMI, pre and post CPAP. (^*^: P value<0.05; ^**^: P value<0.01)

Moreover, the effect of CPAP therapy on blood pressure and lipid parameters of patients with BMI≥30 kg/m^2^ was more than patients with BMI<30 kg/m^2^ (Figure 1).

## DISCUSSION

The results suggested CPAP therapy of OSA patients for 8 weeks statistically led to a significant increase in HDL level and a significant decrease in LDL. On the other hand, Cholesterol, TG and FBG showed no considerable improvement.

Considering the controversial results in previous studies, the effect of CPAP therapy on lipid profile should be clarified. Some studies have pointed out extraordinarily significant increases in lipid and serum lipoprotein levels induced by CPAP or Bi-level Positive Airway Pressure (BiPAP) therapy (15). On the other hand, others suggested no change in lipid profile of patients after one night (22), two nights (23) and/or four months (24) of treatment. Sharma et al. suggested no significant improvement in lipid profile of patients after 3 months using CPAP therapy (25) and similarly another study reported the same result after 12 weeks (26). However, some studies demonstrated the positive effects of CPAP therapy on lipids of patients (21, 27–29). Therefore, taking into account the different results of studies with various durations, it is doubtful if changes in lipid profiles only depend on short-term or long-term therapies. It is seemed that confounding factors such as obesity, physical activity and OSA severity of patients in different studies can play the important role.

Only total cholesterol level showed a significant increase in moderate OSA patients. Following division of patients in terms of Body Mass Index (BMI), it clarified that CPAP therapy was more effective in obese patients than others. The CPAP therapy was more effective in TG and total cholesterol of patients with BMI ≥30 kg/m^2^ than those with BMI <30 kg/m^2^ and it had still led to improvements of HDL and LDL in both groups of patients.

Ishida et al. reported that using CPAP therapy for moderate and severe OSA patients showed no positive effect on inflammatory markers and adiponectin (30). Another study indicated improvements in the modulation of lipidemic factors following adjustment of agents such as obesity and OSA severity (14). Indeed, many studies have mentioned existence of the association of OSA and obesity with dyslipidemia or metabolic syndrome components (8, 14, 31, 32); accordingly, effects of this therapy can be associated with clinical characteristics of patients. As a conclusion, it may be assumed that resolving recurrent hypoxia and sympathetic stimulation of patients could be accompanied with improvements in lipid profile (17).

Furthermore, the results of current study suggested considerable decreases in systolic and diastolic blood pressure levels. In this regard, there is a great deal of evidence in humans and animals indicating the association between OSA and hypertension (33, 34). Hypertension in OSA patients is known as a multifactorial phenomenon and may be associated with factors such as sympathetic overactivity, systemic inflammation, oxidative stress, vasoactive endogenous factors, and endothelial dysfunction (35).

In line with this study, the role of CPAP therapy for 4, 6 or 8 weeks in improvements of systolic and/or diastolic blood pressure has been widely reported (18, 19, 22). But in contrary, many investigators have considered CPAP therapy as a slightly effective treatment in their meta-analysis studies, despite significant effects of CPAP therapy on lipid profile of patients (36–38). It may be noted that CPAP therapy can decrease blood pressure with reducing endothelial inflammation and oxidative stress in OSA (39). But it can be stated that this effect depends on the duration of treatment, and hence, no significant decrease in blood pressure has been recognized in some studies.

Some limitations in many previous studies as well as this study including short-term CPAP therapy, small sample size, uncontrolled physical activities and diets and population characteristics of patients, can lead to no response to CPAP therapy. Accordingly, we attempted to use CPAP for all patients using a uniform protocol and to ensure that no patient has taken antihypertensive or lipid-lowering drugs. It requires further studies with larger sample sizes and longer periods of treatment as well as controlled diet and physical activity. Also, further studies can evaluate the effect of CPAP therapy on OSA patients with and without metabolic syndrome in order to respond to questions such as whether early CPAP therapy, before development of disease or metabolic syndrome, can be more effective in lipid profile and levels of blood pressure (metabolic syndrome components) or not?

## CONCLUSION

The results indicated that CPAP can be effective in improvement of systolic and diastolic blood pressure levels of patients and it can result in increased HDL as well as decreased LDL significantly. However, changes in other lipid parameters were insignificant. The effect of this therapy was more obvious in obese patients (BMI ≥30 kg/m^2^) than others, but it showed no significant difference between patients with moderate OSA and those with severe OSA. So, CPAP therapy could have preventive effect on cardiovascular complications in addition to treatment of OSA.
